# Conversion between the numerical simulation and the calculation using the Deformation Energy Ratio approach

**DOI:** 10.1016/j.mex.2025.103221

**Published:** 2025-02-15

**Authors:** Kirill Golubiatnikov, František Wald

**Affiliations:** Czech Technical University in Prague, Faculty of Civil Engineering, Thakurova 7, 166 29 Prague, Czech Republic

**Keywords:** Structural steel, Numerical simulation, Numerical calculation, Conversion, Deformation energy, Limit values, The Deformation Energy Ratio approach

## Abstract

In numerical simulations and calculations, material curves are defined in different ways. A direct transfer of quantities between them is inaccurate, requiring a proper transformation of key values. The Deformation Energy Ratio approach is based on well-established mechanical principles, such as Neuber's rule and the Equivalent Strain Energy Density method, which express deformation energy as a function of stress and strain. This approach accounts for material conditions in both analyses and enables a more precise limit adjustment. Its simplicity allows for application in both analytical and numerical solutions.

• Allows conversion of values between numerical simulation and calculation for any steel.
• It takes into account the influence of material properties, thus comprehensively considering the effects of different factors.
• The verification has presented a high accuracy of the values obtained. The average deviation is <5.0 % and the maximum deviation is 5.6 %.

## Graphical abstract

**Figure:**
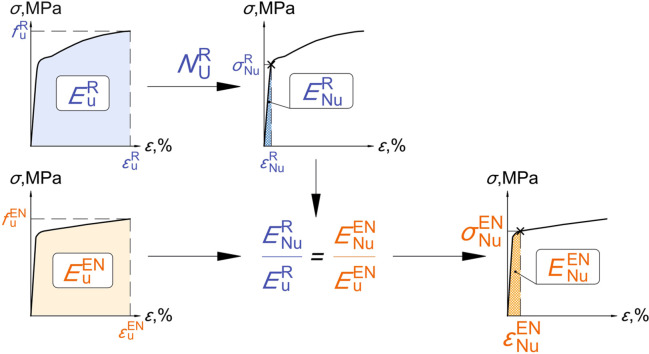

Specifications table

**Table:** 

Subject area:	Engineering
More specific subject area:	Steel structures
Name of your method:	The Deformation Energy Ratio approach
Name and reference of original method:	H. Neuber. Theory of stress concentration for shear-strained prismatical bodies with arbitrary nonlinear stress–strain law. J. Appl. Mech. 28 (1961) 544. https://doi.org/10.1115/1.3641780. K. Molski, G. Glinka. A method of elastic–plastic stress and strain calculation at a notch root. Mater. Sci. Eng. 50 (1981) 93–100. https://doi.org/10.1016/0025–5416(81)90,089–6.
Resource availability:	None.

## Background

For numerical analysis, it is essential to determine limit values, such as the plastic strain limit. Using values derived solely from numerical simulation is inaccurate, as this approach does not account for material property effects. The Deformation Energy Ratio approach facilitates the conversion of values between numerical simulation and numerical calculation, ensuring greater accuracy.

## Method details

### Introduction

The numerical calculation of steel structures using the Finite Element Method requires the use of a limit, which can be defined as the maximum permissible plastic strains $\varepsilon_{\text{Nu}}$, Criterium C2 in [1]. This criterion indicates the value of plastic strains at the design ultimate resistance of the net cross-section $N_{U}$ and represents the safety of member. Generally, the limit of plastic strains $\varepsilon_{\text{Nu}}$ can be represented as a function of two components:

(1) $$\varepsilon_{\text{Nu}}=f\left\{N_{U},\left[f_{y},\;f_{U},\;\varepsilon_{u}\right]\right\}$$

For specimens with weakened cross-sections, such as a hole for a bolt or a reduced width of a beam flange for access to machine maintenance the design ultimate resistance $N_{U}$ represents the force at the onset of the necking part [2] is:

(2) $$N_{U}=\left(k/\gamma_{M2}\right)A_{\text{net}}f_{U}$$

where $A_{\text{net}}$ is the net cross-sectional area, $f_{U}$ is the ultimate strength, $\gamma_{M2}$ is the partial safety factor and k is the reduction factor. The reduction factor is in [2] taken as k=0,9 for rough holes and k=1 for smooth holes. The vales $A_{\text{net}}$ and k are independent of other variables.

The component $\left[f_{y},\;f_{U},\;\varepsilon_{u}\right]$ represents the material model used in the numerical analysis.

**Fig. 1:**
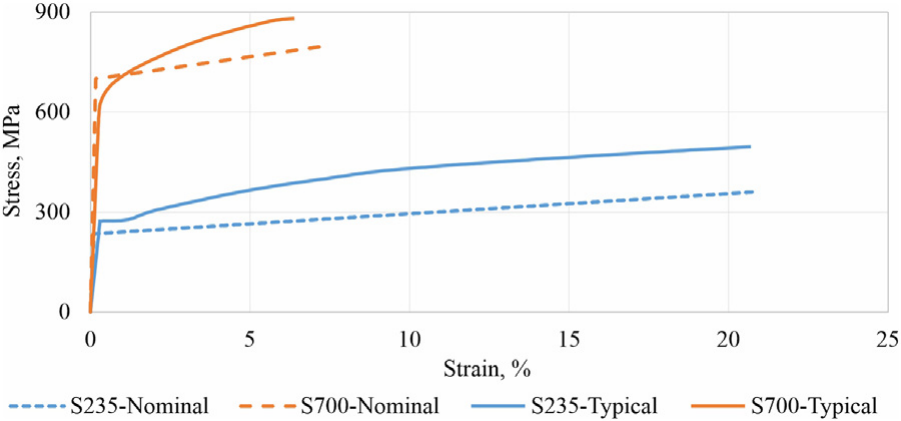
Typical real [5] and nominal [2] material models for steels S235 and S700.

Numerical analysis in steel structures is categorized into numerical simulation and numerical calculation. Numerical simulation (NS) aims to model the mechanical behavior of a specimen as accurately as possible. The material model is defined in the True stress–True strain format with the real material properties such as yield strength $f_{y}^{R}$, ultimate strength $f_{U}^{R}$ and ultimate strain $\varepsilon_{U}^{R}$, without applying a safety factor $\gamma_{M2}$. Typical real material models for S235 and S700 steel grades are shown in Fig. 1. The distributions of mechanical properties are presented for the European steels in [3] and satisfy the structural steel condition $f_{U}/f_{y}\geq 1.1$ z [2].

Numerical calculation provides values relevant to practical engineering applications, representing statistically guaranteed values with a 0.12 % probability of worse outcomes [4]. This type of numerical analysis employs simplified material curves in the Engineering stress–Engineering strain format, incorporating nominal values for yield strength $f_{y}^{\text{EN}}$, ultimate strength $f_{U}^{\text{EN}}$, ultimate strain $\varepsilon_{U}^{\text{EN}}$, along with the partial safety factor $\gamma_{M2}$. Examples of nominal material models are shown in Fig. 1.

**Fig. 2:**
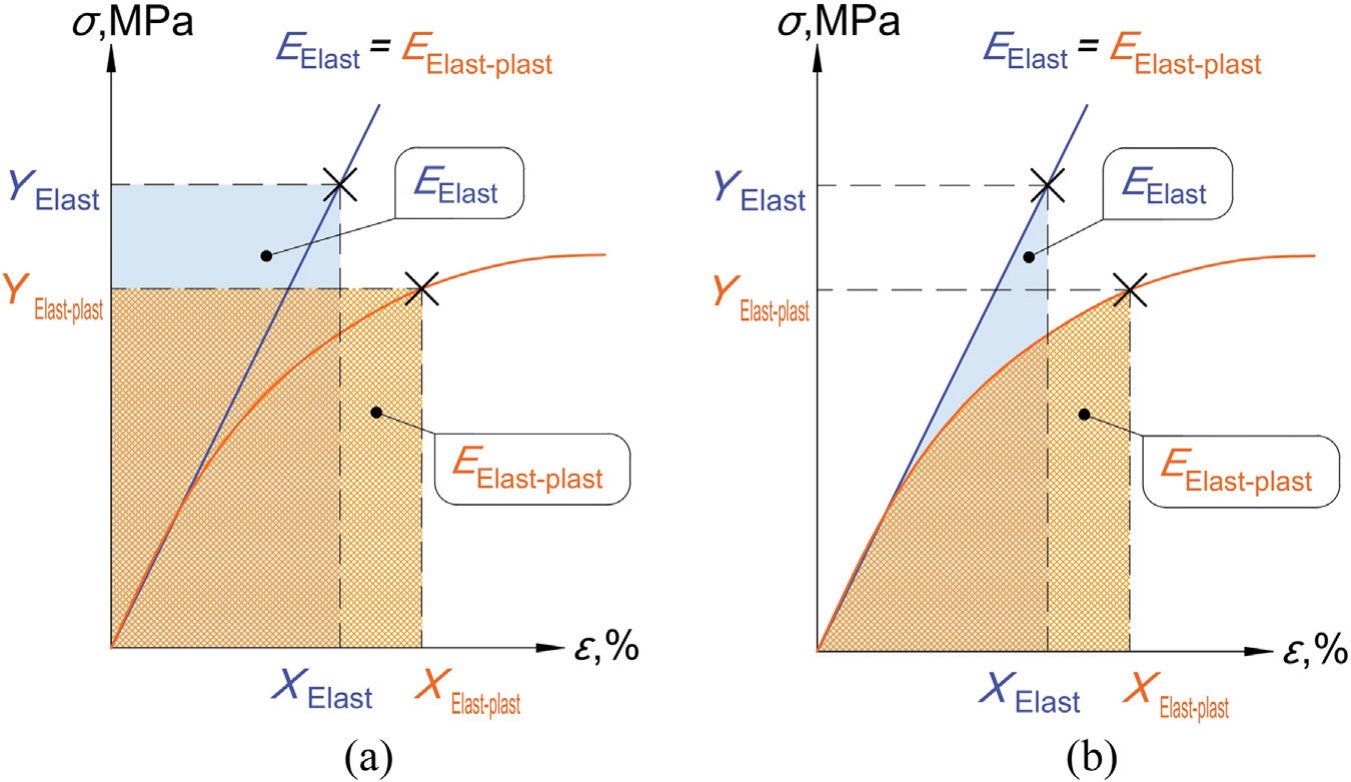
Deformation energy [8] according to a) Neuber rule, b) The Equivalent Strain Energy Density concept.

The general function (1) shows that the plastic strain limit $\varepsilon_{\text{Nu}}$ for numerical simulations requires conversion for numerical calculations. The conversion of values between different types of numerical analysis is analogous to the problem in mechanics of converting elastic calculations to elastoplastic ones. The first solution was proposed by Neuber in [6]. The deformation energy required to deform a sample to *X* mm under nominal stress *Y* MPa can be represented as the area of a rectangle with sides *X* and *Y*, see Fig. 2a). When using a perfectly elastic or elastoplastic material, the absolute amount of energy remains the same, but the ratios of force and deformation change. Glinka and Molski in [7] demonstrated that the concept of energy as a rectangle overestimates elastoplastic behavior, and it is more correct to use only the area under the curve, The Equivalent Strain Energy Density concept (ESED), see Fig. 2b). A similar approach was applied to convert the limit values between two types of numerical analysis.

### Method details

The main objective is to obtain the limit of plastic strain for numerical calculation $\varepsilon_{\text{Nu}}^{\text{EN}}$ for a weakened sample with a net cross-section area $A_{\text{net}}$, which was made from steel with mechanical properties $\left[f_{y}^{R},\;f_{U}^{R},\;\varepsilon_{U}^{R}\right]$ and nominal mechanical properties $\left[f_{y}^{\text{EN}},\;f_{U}^{\text{EN}},\;\varepsilon_{U}^{\text{EN}}\right]$ according to [2]. The first step is to carry out numerical simulation and numerical calculation. Numerical models must satisfy the conditions for numerical analysis of [1]. The result of the numerical analyses will be two stress-strain graphs. The last points ($f_{U}^{R},\;\varepsilon_{U}^{R}$) and ($f_{U}^{\text{EN}},\varepsilon_{U}^{\text{EN}}$) show the moment of reaching the strength limit at least at one point, the beginning of the necking part. The areas under the two graphs, $E_{U}^{R}$ and $E_{U}^{\text{EN}}$, represent the total deformation energies.

The real value of the design ultimate resistance $N_{U}^{R}$ can be determined using Eq. (2). The corresponding values of stress $\sigma_{\text{Nu}}^{R}$ and strain $\varepsilon_{\text{Nu}}^{R}$ are clear from the results of the numerical simulation. The area under the NS graph $E_{\text{Nu}}^{R}$ represents the amount of deformation energy that was used to achieve the design ultimate resistance $N_{U}^{R}$ by the sample.

Since the numerical simulations and the numerical calculations represent the same physical process and differ only in the material curve used, the ratios of the deformation energy of the design ultimate resistance to the total deformation energy should be the same in both cases, as shown in Eq. (3).

(3) $$E_{\text{Nu}}^{R}/E_{u}^{R}=E_{\text{Nu}}^{\text{EN}}/E_{u}^{\text{EN}}\;\to \;E_{\text{Nu}}^{\text{EN}}=\left(E_{\text{Nu}}^{R}\;E_{u}^{\text{EN}}\right)/E_{u}^{R}$$

The value $E_{\text{Nu}}^{\text{EN}}$ allows to find the limit values of the design ultimate resistance $N_{U}^{\text{EN}}$ and the plastic strains $\varepsilon_{U}^{\text{EN}}$ for numerical calculation by an iterative method.

**Fig. 3:**
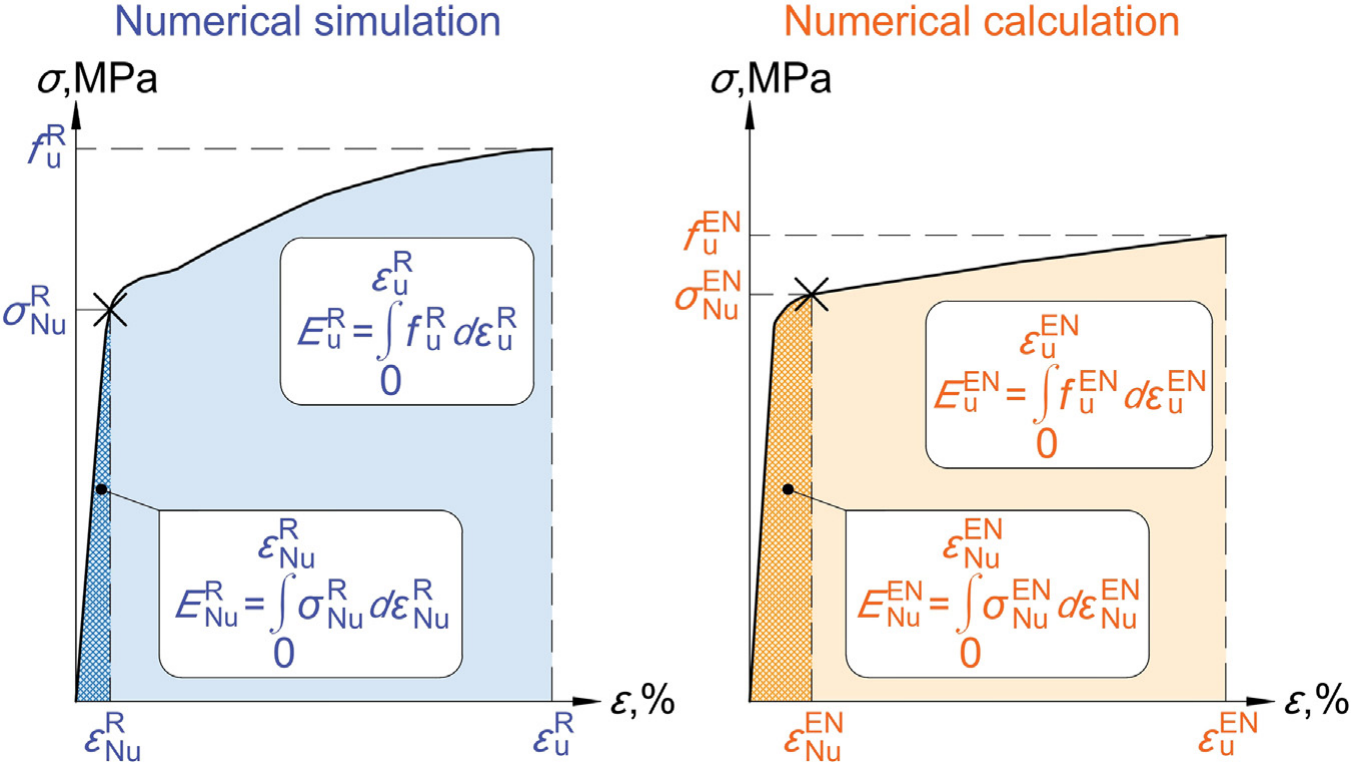
Explanation of the Deformation Energy Ratio approach from numerical simulation to numerical calculation.

### Method validation

The Deformation Energy Ratio approach was verified on samples with two notches from S235 steel. The design ultimate resistances $N_{U}^{\text{EN}}$ for numerical calculation were determined using the proposed approach, and the obtained values were compared with those recommended by prEN 1993–1–1, which were calculated using Eq. (2).

The minimum notch depth d was determined based on the validity condition for using the design ultimate resistance $N_{U}^{\text{EN}}$ of the weakened section with nominal mechanical properties, which must be less than the design plastic resistance $N_{\text{Pl}}^{\text{EN}}$ of the gross cross-section:

(4) $$N_{\text{Pl}}^{\text{EN}}=Af_{y}/\gamma_{M0}\geq N_{U}^{\text{EN}}\to \;d/w=0.06$$

here the partial safety factor $\gamma_{M0}$ is 1. The maximum notch depth was determined based on the permitted bolted hole diameter according to EN 1993–1–8 [9]. If the plate has only one bolted hole, its width w would be equal to only twice the edge distance $e_{2}$. The edge distance is defined in the standard as a function of the hole diameter, with a minimum value of 1.2d. Based on this, the maximum notch depth is:

(5) $$w=2e_{2}\;\to d/w=0.208\approx 0.2$$

The notch radius varied as well. Its values were established based on the stress concentration factor *SCF* under yield strength. This factor represents the ratio of maximum to nominal stress and, in this work, was set to 2, 2.5, 3, or 4. Smaller *SCF*s corresponded to gradual cross-section changes, while larger *SCF*s represented abrupt changes. The length of the free sections was set at 120 mm to ensure that the boundary conditions did not affect the stress at the notch.

**Fig. 4:**
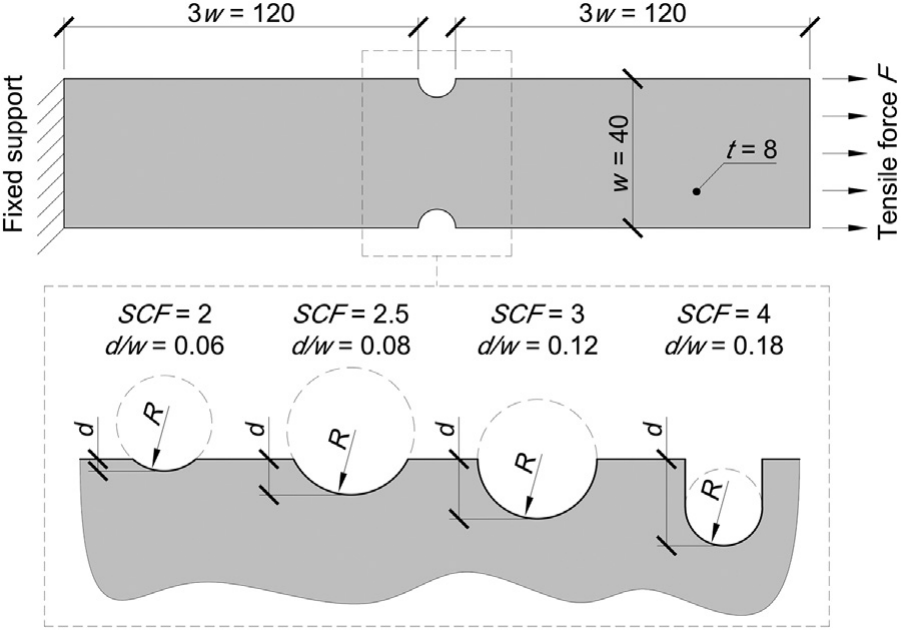
Geometry and boundary conditions of numerical models.

The numerical analyses were conducted using Ansys 2022 R2. Numerical simulations were performed with the typical real material model, while numerical calculations were carried out using the nominal material model, as shown in Fig. 1, for each selected geometry. The mesh was generated using 20-node solid elements with a density of 2 mm × 2 mm, similar to the approach in [10] and [11].

**Fig. 5:**
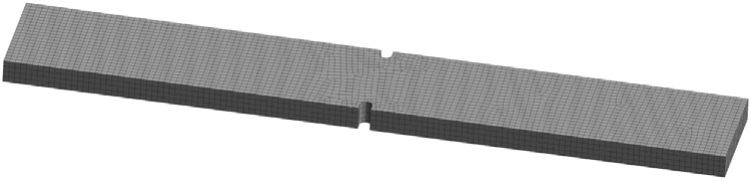
Example of meshing.

**Fig. 6:**
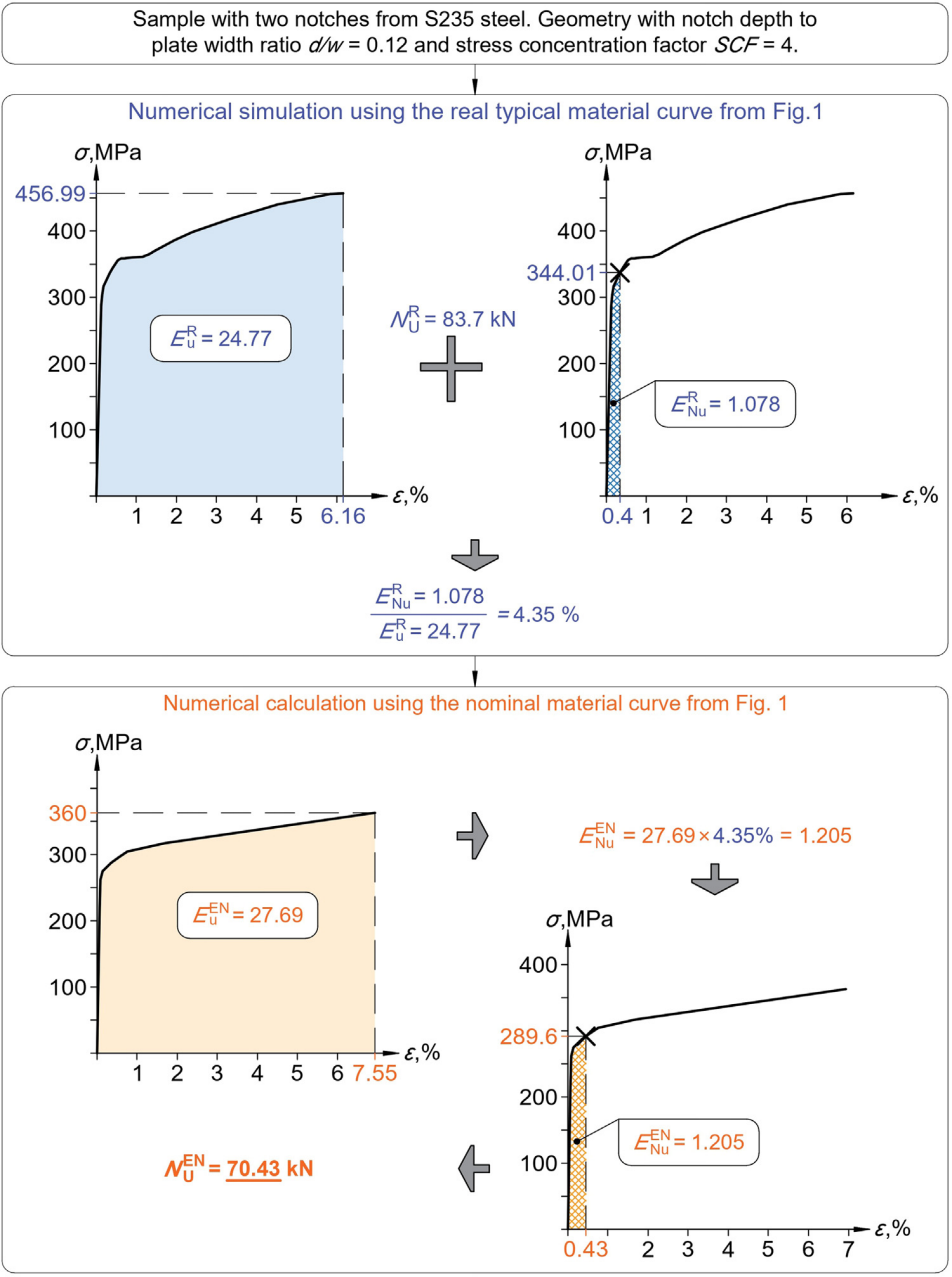
Flowchart of the design ultimate resistance determination using the Deformation Energy Ratio approach for a sample with a notch depth to plate width ratio of 0.12 and a stress concentration factor of 4.

An example of the design ultimate resistance determination using the Deformation Energy Ratio approach for a sample with a notch depth-to-plate width ratio of 0.12 and a stress concentration factor of 4 is shown in Fig. 6. The stress-strain graph for the numerical simulation was obtained through numerical analysis. The area under the graph represents the total deformation energy $E_{U}^{R}=24.77$. The design ultimate resistance for the numerical simulation $N_{U}^{R}=83.7$ kN was calculated using Eq. (2). The deformation energy of the design ultimate resistance is represented by an area limited by the stress $\sigma_{\text{Nu}}^{R}=344.01$ MPa and strain $\varepsilon_{\text{Nu}}^{R}=0.4$ %. The design ultimate resistance was reached at 4.35% of the total deformation energy. The total deformation energy in the numerical calculation $E_{U}^{\text{EN}}=27.69$ was determined similarly. The Deformation Energy Ratios must be equal in both analyses. Based on this condition, the deformation energy of the design ultimate resistance for numerical calculation $E_{\text{Nu}}^{\text{EN}}$ equals to 1.205. In the final step, the limiting stress and strain were determined using an iterative method, $\sigma_{\text{Nu}}^{\text{EN}}=289.6$ MPa and $\varepsilon_{\text{Nu}}^{\text{EN}}=0.43$ %. The corresponding force $N_{U}^{\text{EN}}=70.43$ kN is the design ultimate resistance for numerical calculation and is almost identical to the recommended resistance according to Eq. (2), i.e. 70.04 kN.

A total of 40 samples were analyzed using the Deformation Energy Ratio approach to determine the design ultimate resistances $N_{U}^{\text{EN}}$ for numerical calculation. The obtained values were compared with the recommended analytical resistances according to EN 1993-1-1 [2] using Eq. (2). The verification results demonstrate that the proposed Deformation Energy Ratio approach determines the limit values with sufficient accuracy. The maximum deviation from the analytical value is 5.6 %, occurring for the smallest notch. The accuracy improves with larger and more critical notches. The average deviation is less than 5 %.

**Fig. 7:**
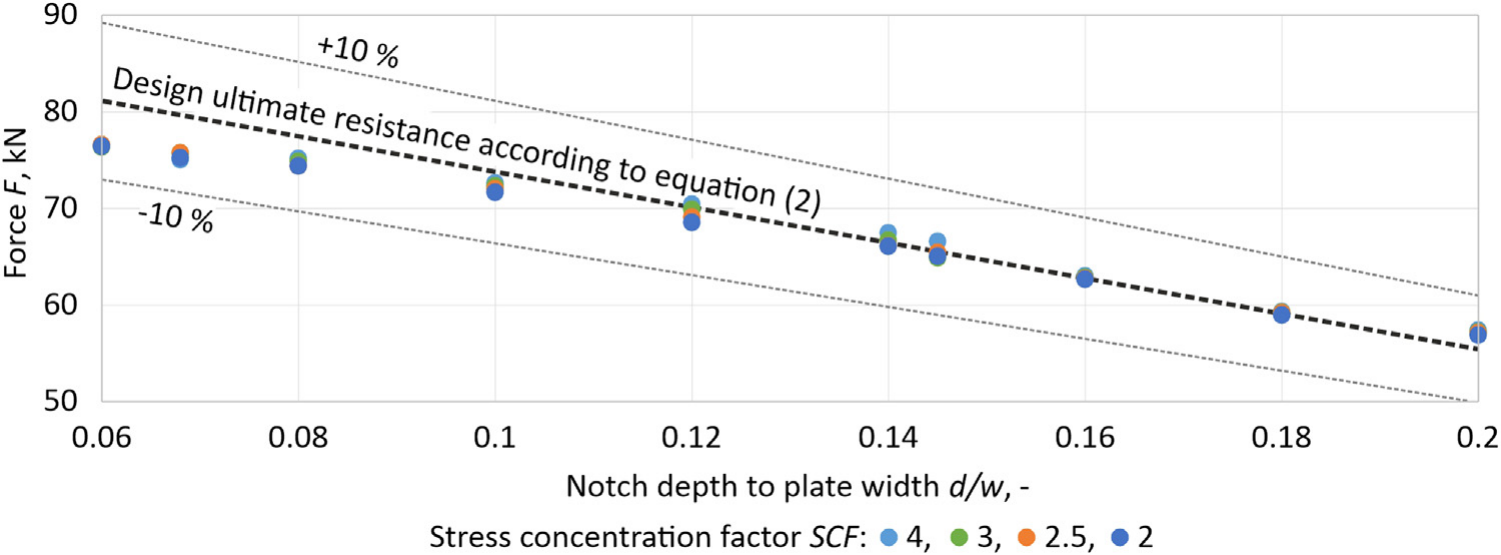
Verification of the Deformation Energy Ratio approach on steel S235.

## Conclusion

In this paper, the Deformation Energy Ratio approach for converting values from numerical simulation to numerical calculation was proposed. This approach considers different material models used in both analyses. The deformation energy was represented as a function of stress and strain, similar to the Equivalent Strain Energy Density concept and Neuber's rule. This approach is universal and applicable to any type of steel. The Deformation Energy Ratio approach can be employed with both analytical and numerical analyses. Its validity was verified against the recommended analytical resistances according to EN 1993–1–1 [2]. The maximum deviation was 5.6 %, occurring for the smallest notch, while the average deviation remained below 5 %. However, the accuracy improved for larger and more critical notches.

## CRediT authorship contribution statement

**Kirill Golubiatnikov:** Conceptualization, Formal analysis, Methodology, Validation, Writing – original draft. **František Wald:** Writing – review & editing, Supervision, Funding acquisition, Project administration.

## Declarations of competing interest

The authors declare that they have no known competing financial interests or personal relationships that could have appeared to influence the work reported in this paper.

## Data Availability

No data was used for the research described in the article.
